# Additional Insertion of gC Gene Triggers Better Immune Efficacy of TK/gI/gE-Deleted Pseudorabies Virus in Mice

**DOI:** 10.3390/v16050706

**Published:** 2024-04-29

**Authors:** Zhuoyun Wu, Jiahuan Deng, Meijing Chen, Peiqi Lu, Zhibin Yan, Xiaoyan Wu, Qiuyun Ji, Huiying Fan, Yongwen Luo, Chunmei Ju

**Affiliations:** Key Laboratory of Zoonosis Prevention and Control of Guangdong Province, College of Veterinary Medicine, South China Agricultural University, Guangzhou 510642, China; wzzy0615@stu.scau.edu.cn (Z.W.); 20213073021@stu.scau.edu.cn (J.D.); mjchen@bfrbiotech.com.cn (M.C.); 20223073086lu@stu.scau.edu.cn (P.L.); 18826271402@163.com (Z.Y.); 18236909354@163.com (X.W.); 20193073034@stu.scau.edu.cn (Q.J.); fanhy@scau.edu.cn (H.F.)

**Keywords:** pseudorabies virus, recombinant virus, attenuated vaccine, immune efficacy

## Abstract

In recent years, pseudorabies virus (PRV) variants have resulted in an epidemic in swine herds and huge economic losses in China. Therefore, it is essential to develop an efficacious vaccine against the spread of PRV variants. Here, the triple-gene-deletion virus and the triple-gene-deletion plus gC virus were constructed by homologous recombination (HR). And then, their growth capacity, proliferation ability, and immune efficacy were evaluated. The results showed that the growth kinetics of the recombinant viruses were similar to those of the parental strain PRV-AH. Compared with the triple-gene-deletion virus group, the more dominant level of neutralizing antibody (NA) can be induced in the triple-gene-deletion plus gC virus group with the same 10^6.0^ TCID_50_ dose after 4 and 6 weeks post-initial immunization (PII) (*p* < 0.0001). In addition, the antibody titers in mice immunized with the triple-gene-deletion plus gC virus were significantly higher than those immunized with triple-gene deletion virus with the same 10^5.0^ TCID_50_ dose after 6 weeks PII (*p* < 0.001). More importantly, in the triple-gene-deletion plus gC virus group with 10^5.0^ TCID_50_, the level of NA was close to that in the triple-gene deletion virus group with 10^6.0^ TCID_50_ at 6 weeks PII. Meanwhile, the cytokines IL-4 and IFN-γ in sera were tested by enzyme-linked immunosorbent assay (ELISA) in each group. The highest level of IL-4 or IFN-γ was also elicited in the triple-gene deletion plus gC virus group at a dose of 10^6.0^ TCID_50_. After challenge with PRV-AH, the survival rates of the triple-gene deletion plus gC virus immunized groups were higher than those of other groups. In immunized groups with 10^5.0^ TCID_50_, the survival rate shows a significant difference between the triple-gene deletion plus gC virus group (75%, 6/8) and the triple-gene deletion virus group (12.5%, 1/8). In general, the immune efficacy of the PRV TK/gI/gE-deleted virus can be increased with additional gC insertion in mice, which has potential for developing an attenuated vaccine candidate for PRV control.

## 1. Introduction

Pseudorabies (PR), also known as Aujeszky’s disease, is caused by the pseudorabies virus (PRV) [1]. Since the discovery of PRV in 1902, it has been regarded as one of the most important pathogens, causing serious economic losses to the pig industry around the world [2,3,4]. PRV is classified within the family *Herpesviridae*, subfamily *Alphaherpesviridae*, and belongs to the genus Varicella virus [5]. Although swine are the only natural host of PRV, numerous domestic animals and wildlife species can also be infected [5,6,7,8,9,10,11,12]. Furthermore, more than 20 cases of human PRV infection have been reported since 2018. Workers in the pig industry are most likely to be infected by PRV directly or indirectly through conjunctiva, skin wounds, and syringe stab wounds [13]. Therefore, we need to endeavor to develop effective strategies, such as vaccines and anti-viral agents, for the control of PRV.

PRV infection can cause fever, neurologic symptoms, reproductive failure, and respiratory symptoms [14]. Swine manifest different clinical symptoms depending on their distinct ages. Severe neurological symptoms and death occur mainly in newborn and suckling pigs; respiratory symptoms and growth retardation in growing pigs; and reproductive disorders in sows [15,16,17]. PRV can also establish latent infection in the peripheral nervous system (PNS) of pigs and be reactivated when stimulated by stressors, which brings great difficulties to the control and eradication of pseudorabies [13].

Vaccination is the main strategy to prevent animals from contracting PRV. Bartha-K61 is an attenuated strain in which the complete gE gene and the partial gI gene have been deleted [18,19]. A vaccine based on the Bartha-K61 strain has played a critical role in the control of PR since the 1990s [3]. However, the new PRV mutant strains have been prevalent in pig farms since 2011 due to the differences in pathogenicity and immunogenicity between variants [20,21,22,23,24]. Therefore, it is a viable strategy to develop vaccines based on emerging PRV variants [25]. Attenuated and inactivated vaccines are often used to control the spread of PRV. Moreover, the attenuated vaccines generally show better immune efficacy, which can induce higher levels of neutralizing antibodies and T cell-mediated immunity [26,27].

PRV is a double-stranded DNA virus of approximately 142 kb in length, and the genome consists of a unique long region (UL), a unique short region (US), internal repetitive sequences (IRS), and terminal repetitive sequences (TRS) [17]. Glycoproteins, as the surface structure of the virion-contacting cell, are the host’s major targets of immune defense [17]. There are 11 glycoproteins in the membrane of PRV, including gB, gC, gD, gE, gG, gH, gI, gK, gL, gM, and gN [1].

Glycoproteins gE and gI are required for virus virulence and spread but are non-essential for viral replication [17]. The thymidine kinase (TK) gene is the major virulence gene of PRV. The neurotoxicity of PRV can be weakened if the integrity of the TK gene is disrupted [28]. Therefore, we aimed to develop an attenuated virus with gene deletions of gE, gI, and TK based on the mutant strain PRV-AH [26,29]. The glycoprotein gC is one of the main antigens that induce neutralizing antibodies, and also acts as a target for cytotoxic T lymphocytes [30,31]. Amino acids at position 1~22 are the signal peptides that help gC localize in the cell membrane. The anchoring region of gC on the membrane is at the position of 436~470 amino acids. The region of 23~435 amino acids plays an important role in the production of neutralizing antibodies against PRV [17,32]. Hence, an additional gC gene was considered to be added to the TK/gE/gI-deleted virus to enhance humoral immunity and cell-mediated immunity.

In this study, we constructed the triple-gene deletion virus and the triple-gene deletion plus gC virus recombinant strain based on a variant PRV which was isolated from the brain of an aborted fetus on a pig farm in Anhui, China [33]. The efficacy of these two recombinant viruses was evaluated, including neutralizing antibodies, cytokines, and protection rate in Kunming mice.

## 2. Materials and Methods

### 2.1. Cells, Viruses, and Plasmid

BHK-21 cells were cultured in Dulbecco’s Modified Eagle’s Medium (Procell, Wuhan China) with additional 10% FBS (Thermo Fisher, Waltham, MA, USA) at 37 °C with 5% CO_2_, and the propagation of PRV was used. The parental virus of the PRV-AH strain had been identified as a mutant PRV [33]. The plasmids pMD-UA-EGFP-DA, which contained the homologous arms of the gE/gI gene and the EGFP expression cassette, and pMD-UA-gC-DA, which contained the homologous arms of the gE/gI gene and the gC-His expression cassette, were generated, respectively, in our previous study [34].

### 2.2. Construction of Transfer Vectors and Recombinant Viruses

**Figure 1:**
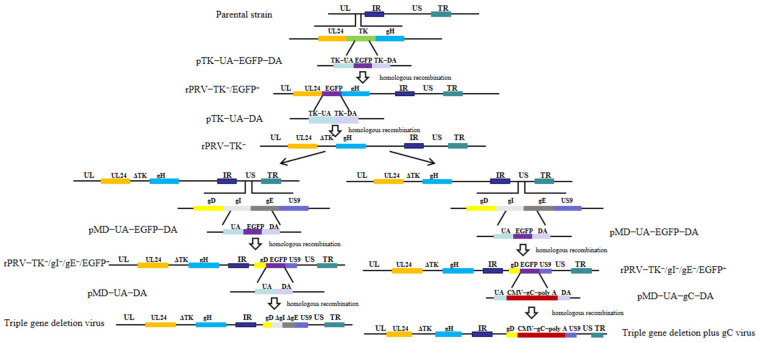
Construction strategies of the triple-gene deletion virus and the triple-gene deletion plus gC virus. First, the TK gene of the parental strain was deleted with EGFP as the selection marker by homologous recombination (HR) twice. Then, EGFP was added at the locations of gI and gE to construct the triple-gene deletion plus EGFP virus. Finally, the triple-gene deletion plus gC virus was produced by HR between the triple-gene deletion plus EGFP virus and pMD-UA-gC-DA. An additional gC expression cassette replaces the deleted gI and gE regions. Similarly, the triple-gene deletion virus was constructed by HR between the triple-gene deletion plus EGFP virus and pMD-UA-DA to delete EGFP.

Homologous recombination (hereinafter, HR) was used to construct the recombinant virus. In short, the homologous up arm (TK-UA) and down arm (TK-DA) of the TK gene were cloned by primers TK-UA-forward/TK-UA-reverse and TK-DA-forward/TK-DA-reverse (Table 1) and inserted into pMD-18T, respectively, to generate pTK-UA-DA. EGFP was obtained by PCR using EGFP-Forward and EGFP-Reverse primers (Table 1) and then linked with pTK-UA-DA to construct the transfer vector pTK-UA-EGFP-DA. Next, BHK-21 cells were transfected with pTK-UA-EGFP-DA using Lipofectamine ^TM^ 2000 (Thermo Fisher, Waltham, MA, USA) and subsequently inoculated with PRV-AH. The recombinant virus with TK deletion and EGFP insertion, named rPRV-TK^−^/EGFP^+^, was produced by HR and purified with EGFP as the selection marker. Similarly, the virus rPRV-TK^−^/EGFP^+^ and the plasmid pTK-UA-DA were used to construct the rPRV-TK^−^ by HR. (Figure 1). Finally, the triple-gene deletion plus gC virus was generated by HR twice in the region of gI and gE to delete gI and gE and insert additional gC (Figure 1).

### 2.3. Plaque Assay

The reproduction of viruses can cause cells to rupture and die. When covered with agarose, the released viruses can only spread from the initial infected cell to neighboring cells, resulting in a plaque. Plaque sizes reflect the reproduction cycle of a virus in a particular cell type. To purify the triple-gene deletion virus or the triple-gene deletion plus gC virus, cells were cultured in 6-well plates, and tenfold serial dilutions of a virus sample were inoculated onto BHK monolayers. After incubating for 60 min and washing three times with PBS, BHK cells were covered with phenol-red-free DMEM containing 2% low-melting-point agarose and incubated for a suitable incubation period. The BHK monolayers were stained with 0.002% neutral red, and plaques were observed.

### 2.4. Growth Kinetics

In order to evaluate the growth ability of the triple-gene deletion virus, the triple-gene deletion plus gC virus, or the parental strain, viruses were inoculated on BHK-21 cells and harvested every 8 h after inoculation. Then, the viral titers were tested, and the kinetics curves were drawn.

### 2.5. Western Blotting Analysis

The His-tag antibody (Beyotime, Shanghai, China) was used to test whether the inserted gC was successfully expressed due to the gC gene linked with His-tag. BHK-21 cells were respectively inoculated with the parental strain, the triple-gene deletion virus, and the triple-gene deletion plus gC virus (MOI = 0.01) and collected at 48 hpi. Proteins were obtained from infected cells using radioimmunoprecipitation assay lysis buffer (Thermo Fisher, Waltham, MA, USA). The same amounts of proteins were separated by 10% SDS-PAGE and subsequently transferred onto PVDF membrane. The membrane was incubated with His-tag antibody, followed by incubation with HRP-labeled secondary antibody (Thermo Fisher, Massachusetts USA). NcmECL Ultra Luminol/Enhancer Reagent (A) and NcmECL Ultra Stabilized Peroxide Reagent (B) were used to display the bands (NCM Biotech, Suzhou, China) and observed by exposure instrument (Tanon, Shanghai, China).

### 2.6. Immunization and Challenge in Mice

**Figure 2:**
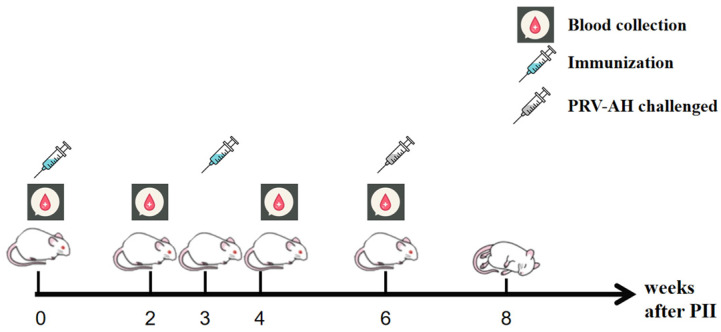
Immune procedure diagram. The mice were randomly divided into 6 groups and immunized at week 0 and week 3. Sera were collected at weeks 0, 2, 4, and 6. Mice were challenged with the PRV-AH strain at week 6, and the survival rates of mice were recorded from week 6 to 8.

Six groups (A–F) of SPF Kunming mice (5 weeks old) were used in the animal experiment, and 8 mice were contained in each group. Group A or Group B were inoculated with the triple-gene deletion plus gC virus (100 µL, 10^6.0^ TCID_50_/100 µL) or the triple-gene deletion virus (100 µL, 10^6.0^ TCID_50_/100 µL) by intramuscular injection. Group C or Group D were inoculated with the two above-mentioned viruses (100 µL, 10^5.0^ TCID_50_/100 µL), respectively, by the same method. Group E was intramuscularly inoculated with 100 µL of HB-98 attenuated commercial vaccine (10^5.5^ TCID_50_/100 µL) (Keqian Biology, Wuhan China). The 10^5.5^ TCID_50_ dose of the HB-98 commercial vaccine used in this study was suggested by the vaccine producer. The two doses of 10^5.0^ TCID_50_ and 10^6.0^ TCID_50_ in the experiment were designed based on our previous studies [34,35,36]. As a negative control, Group F was inoculated with the same amount of DMEM. The immune procedure of this experiment is shown in Figure 2. With the same dose and route, booster immunization was administered in each group at 3 weeks post-initial immunization (PII). The sera were obtained at 0, 2, 4, and 6 weeks PII. After 3 weeks of booster immunization, the mice in each group were intramuscularly infected with the parental strain at a dose of 10^5.5^ TCID_50_. Clinical signs and survival rates in each group were recorded for 2 weeks after the challenge.

### 2.7. Serum-Virus Neutralizing Test (SNT)

NA levels in sera were evaluated by SNT. Sera were inactivated at 56 °C for 30 min and were twofold serially diluted with DMEM. Fifty microliters of diluted sera and the parental strain at a dose of 100 TCID_50_ were added to each well of 96-well plates, and incubated at 37 °C for 1 h. Then, a cell suspension containing about 2 × 10^4.0^ cells (100 µL) was added to each well and incubated at 37 °C. Ultimately, the cytopathic effect (CPE) was observed daily. The Reed–Muench method was used to calculate the NA titers of sera.

### 2.8. Enzyme-Linked Immunosorbent Assay

Antibody titers against PRV were detected with indirect ELISA in the sera of immunized mice. Briefly, PRV-AH virions (purified by ultracentrifugation) were diluted appropriately with carbonate buffer solution to 1:800, which was confirmed by the preliminary experiment, and added to 96-well microplates. The microplates were placed at 37 °C for 1 h, then overnight at 4 °C. The coating solution was removed, and the microplates were washed three times with PBST. Then, 5% skim milk powder was added to the microplates and they were incubated at 37 °C for 2 h. After washing, the sera with a dilution of 1:200 were added to the microplates and incubated at 37 °C for 30 min, followed by incubation with HRP-conjugated goat anti-mouse secondary antibody (Beyotime, Shanghai, China, 1:250) for 30 min at 37 °C. Tetramethylbenzidine (TMB) substrate was added for a color reaction, followed by stopping with 1 mol/L H_2_SO_4_. The optical density (OD) was measured at 450 nm, and a statistical analysis was carried out.

The cytokines IFN-γ and IL-4 in the sera of immunized mice were detected using a commercial mouse cytokine immunoassay ELISA kit (Meimian, Yancheng, China) according to the manufacturer’s instructions.

### 2.9. Statistical Analysis

GraphPad Prism version 8.0 for Windows (GraphPad Software, San Diego, CA, USA) was used for statistical analysis. Pairwise comparisons between different groups were performed using Tukey’s test. The Log-rank (Mantel–Cox) test was used in pairwise comparisons of survival rates between groups. Significant differences of *p* < 0.05, *p* < 0.01, *p* < 0.001, and *p* < 0.0001 were shown as *, **, ***, and ****. Non-significant differences of *p* > 0.05 were shown as ns.

## 3. Results

### 3.1. Identification of the Triple-Gene Deletion Virus and the Triple-Gene Deletion Plus gc Virus

**Figure 3:**
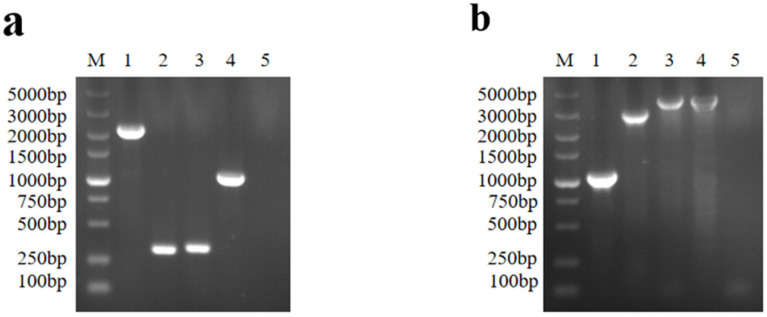
Verification of PRV the triple-gene deletion virus and the triple-gene deletion plus gC virus by PCR. (**a**) Verification of the TK region. M: DNA marker; 1: rPRV-TK^−^/EGFP^+^/gI^−^/gE^−^; 2: the triple-gene deletion virus; 3: the triple-gene deletion plus gC virus; 4: the parental strain; 5: cell control. (**b**) Verification of gI/gE region. M: DNA marker; 1: the triple-gene deletion virus; 2: rPRV-TK^−^/gI^−^/gE^−^/EGFP^+^; 3: the triple-gene deletion plus gC virus; 4: the parental strain; 5: cell control.

TKdet-forward and TKdet-reverse primers were used to detect the TK gene. The PCR results were consistent with expectations. The amplified fragments showed the length of 2159 bp in rPRV-TK^−^/EGFP^+^/gI^−^/gE^−^, 329 bp in the triple-gene deletion virus, 329 bp in the triple-gene deletion plus gC virus, and 1128 bp in the parental strain (Figure 3a).

In the same way, the gEdet-forward and gEdet-reverse primers were used to identify the gI/gE region of the triple-gene deletion virus and the triple-gene deletion plus gC virus by PCR. The amplified fragments showed the length of 1049 bp in the triple-gene deletion virus, 2879 bp in rPRV-TK^−^/gI^−^/gE^−^/EGFP^+^, 3732 bp in the parental strain, and 3897 bp in the triple-gene deletion plus gC virus (Figure 3b). Combined with the sequencing, the results confirmed that the triple-gene deletion virus and the triple-gene deletion plus gC virus were successfully obtained.

### 3.2. Analysis of the Inserted gC in the Triple-Gene Deletion Plus gC Virus by Western Blotting

**Figure 4:**
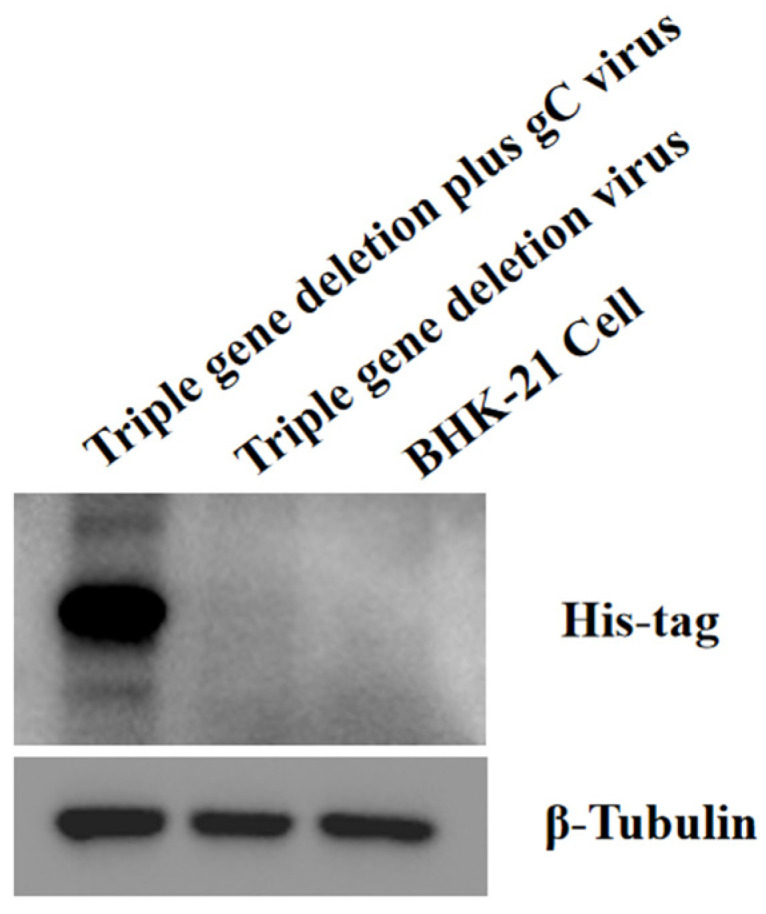
Analysis of gC-His protein by Western blotting. The inserted gC was linked with His-tag; therefore, His-tag McAb could be used for the detection of gC-His fusion protein in cells inoculated with the triple-gene deletion plus gC virus. Three bands were detected with different glycosylation states of the protein in the sample of the triple-gene deletion plus gC virus.

The triple-gene deletion virus and the triple-gene deletion plus gC virus-infected BHK-21 cells, respectively, and total proteins were extracted from infected cells for Western blotting. The result showed that proteins were only expressed in inoculated cells with the triple-gene deletion plus gC virus using His-tag McAb. These bands were in three forms, representing different glycosylation states of the protein. However, no protein band was detected in the cells infected with the triple-gene deletion virus or BHK-21 cells (Figure 4).

### 3.3. The Growth Kinetics of the Triple-Gene Deletion Virus and the Triple-Gene Deletion Plus gC Virus

**Figure 5:**
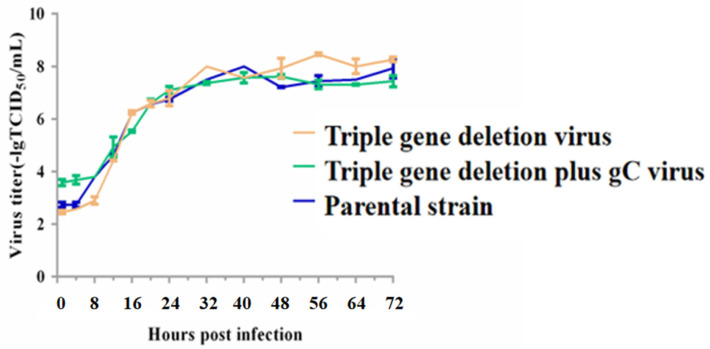
Growth kinetics of the triple-gene deletion virus, the triple-gene deletion plus gC virus, and the parental strain. The yellow, green, and blue lines represent the growth curves of the triple-gene deletion virus, the triple-gene deletion plus gC virus, and the parental strain virus, respectively. There was no significant difference in the growth properties between these strains.

**Figure 6:**
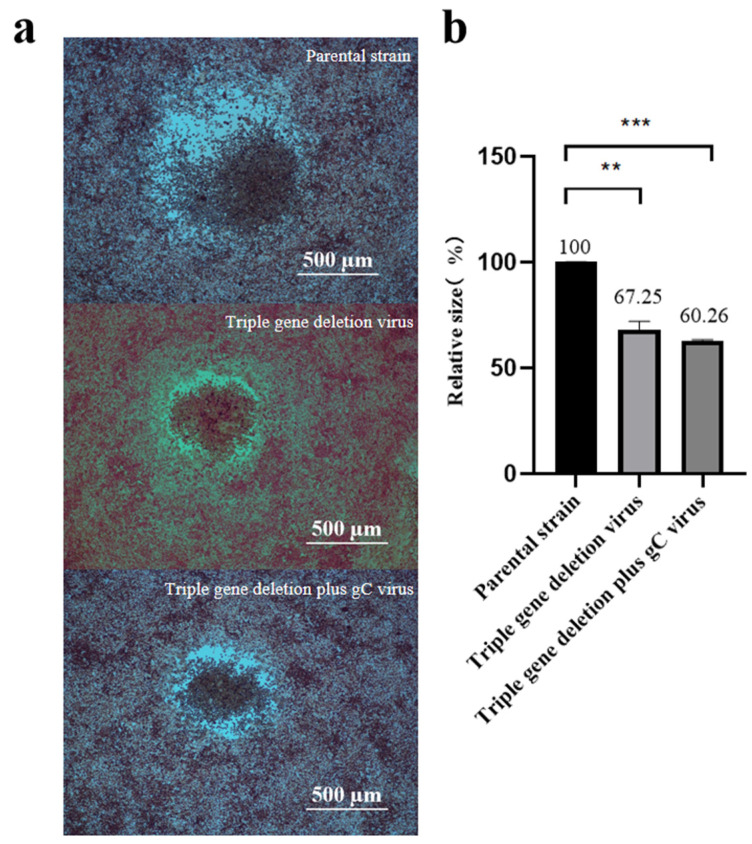
Comparison of plaque morphology and size of the triple-gene deletion virus, the triple-gene deletion plus gC virus, and the parental strain. (**a**) Plaques were stained with neutral red at 72 h after virus infection. (**b**) The average relative size of 20 plaques in each strain is shown as a percentage by setting that of the parental strain to 100% (** *p* < 0.01; *** *p* < 0.001).

The growth kinetics analysis was performed to compare the proliferation properties of the triple-gene deletion virus, the triple-gene deletion plus gC virus, or the parental strain. From the growth curves, the proliferation properties of the triple-gene deletion virus and the triple-gene deletion plus gC virus resembled those of the parental strain (Figure 5). However, the plaque sizes of recombinant viruses were significantly smaller than those of the parental strain (Figure 6).

### 3.4. Antibodies Induced by the Triple-Gene Deletion Virus and the Triple-Gene Deletion Plus gC Virus

**Figure 7:**
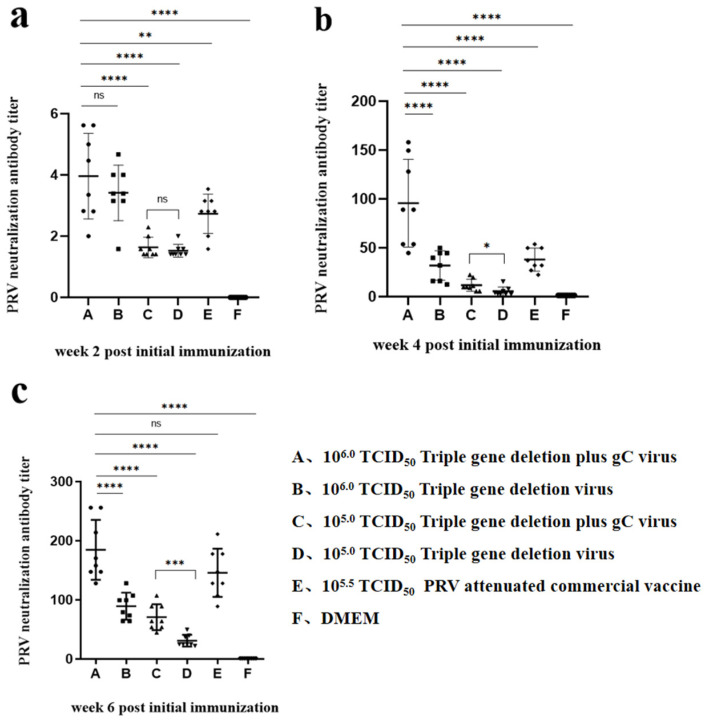
The NA titers of sera in immunized mice. Sera were collected at week 0, 2, 4, and 6 PII to detect NA titers. (**a**) week 2 PII. (**b**) week 4 PII. (**c**) week 6 PII (* *p* < 0.05; ** *p* < 0.01; *** *p* < 0.001; **** *p* < 0.0001). The dots, squares, triangles, and rhombuses represent data for each individual in each group.

Anti-PRV neutralizing antibodies were examined at 0, 2, 4, and 6 weeks PII. As expected, no neutralizing antibodies were detected in mice before immunization. After booster immunization, neutralizing antibody titers increased significantly in each immunized group. The antibody titers immunized with the triple-gene deletion plus gC virus in mice were significantly higher than those immunized with the triple-gene deletion virus at the same 10^5.0^ TCID_50_ dose after 6 weeks PII (*p* < 0.001). At 6 weeks PII, the NA titer was up to 1:184.75 in the triple-gene deletion plus gC virus group with 10^6.0^ TCID_50_, while it was 1:89.45 in the triple-gene deletion virus group with the same dosage. Additionally, compared with the dose of 10^5.0^ TCID_50_, 10^6.0^ TCID_50_ of the triple-gene deletion plus gC virus or the triple-gene deletion virus was able to elicit a higher level of neutralizing antibodies. More importantly, in the triple-gene deletion plus gC virus group with 10^5.0^ TCID_50_, the level of neutralizing antibody was close to that in the triple-gene deletion virus group with 10^6.0^ TCID_50_ at 6 weeks PII (Figure 7).

**Figure 8:**
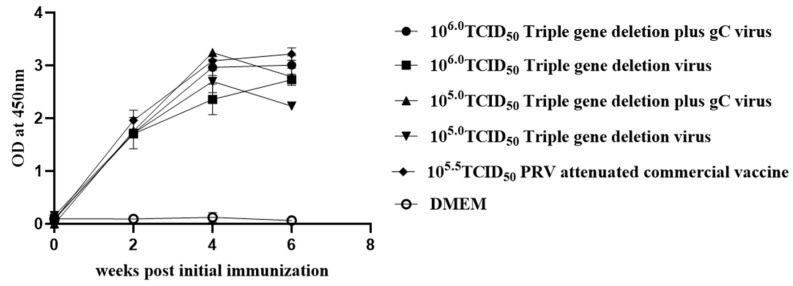
The PRV-specific IgG were tested by indirect ELISA in the sera of immunized mice at 0, 2, 4, and 6 weeks PII. The microplate was coated with PRV-AH virions, and the sera were diluted at a ratio of 1:200. TMB was used as the substrate, and the reaction was terminated with 1 mol/L H_2_SO_4_. The value of OD_450nm_ was measured in each diluted serum.

PRV-specific IgG antibodies were tested by ELISA in the sera of mice at the same time-points PII. The IgG titers increased gradually, and the peak was at week 4 or week 6 PII. Except for the DMEM blank control group, there were no significant differences among the immunized groups (Figure 8). A downward trend of OD values at 450 nm appeared in both 10^5.0^ TCID_50_ dose groups from week 4 to week 6, which revealed that IgG antibodies maintained a short duration at low immunization doses.

### 3.5. Detection of Cytokines IFN-γ and IL-4 in the Sera of Immunized Mice

**Figure 9:**
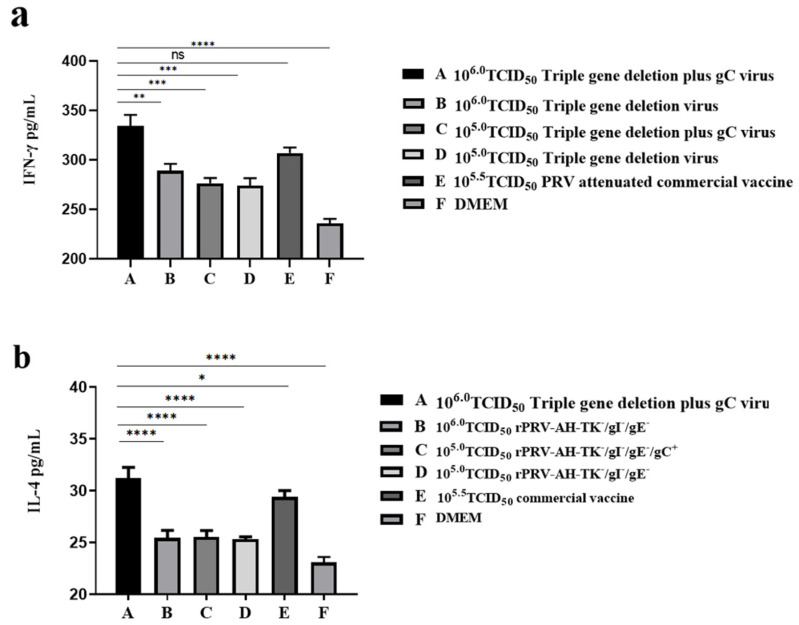
The cytokines IFN-γ and IL-4 were tested by ELISA in the sera of immunized mice at week 6 PII (* *p* < 0.05; ** *p* < 0.01; *** *p* < 0.001; **** *p* < 0.0001, ns *p* > 0.05). The linear regression equation of the standard curve is obtained by using the concentration and OD value of the standard substance, and the OD value of the sera sample is substituted into the equation to calculate the concentration. The actual concentration of the IFN-γ or IL-4 in the serum sample was determined by multiplying the dilution ratio. (**a**) The concentration of IFN-γ in the sera of immunized mice. (**b**) The concentration of IL-4 in the sera of immunized mice.

IFN-γ is important for protective PRV-specific immunity, which can elicit Th1-type cellular immunity and act on macrophages and NK cells to improve natural immunity. The Th2 cytokine IL-4 can activate B cells and promote the proliferation of T cells. Therefore, the cytokines IFN-γ and IL-4 were measured by ELISA in the sera of immunized mice at week 6 PII. Obviously, the expression levels of IFN-γ and IL-4 in the triple-gene deletion plus gC virus group with 10^6.0^ TCID_50_ were much higher than those in other groups, except the commercial vaccine group (Figure 9).

### 3.6. Protection Efficacy of the Triple-Gene Deletion Virus and the Triple-Gene Deletion Plus gC Virus

**Figure 10:**
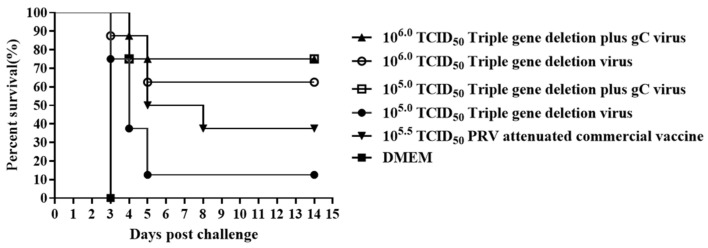
The survival rate curves of mice infected with the parental strain at week 6 PII. After the challenge, time of death for mice was recorded for 14 days, and subsequently, the survival rate curve for each group was drawn.

At 6 weeks PII, all groups were intramuscularly challenged with 10^5.5^ TCID_50_ of the parental strain. All mice died in the DMEM control group and displayed typical PRV neurological symptoms before death. In the groups immunized with 10^6.0^ TCID_50_, the survival rates of the triple-gene deletion plus gC virus group and the triple-gene deletion virus group reached 75% (6/8) and 62.5% (5/8), respectively. In the groups immunized with 10^5.0^ TCID_50_, the survival rate of the triple-gene deletion plus gC virus group was 75% (6/8), while that of the triple-gene deletion virus group was only 12.5% (1/8) (Figure 10). According to the findings, at high immunization doses, there was no significant difference in the protection rate between the triple-gene deletion plus gC virus group and the triple-gene deletion virus group (ns, *p* > 0.5). However, at low immunization doses, it was significantly higher in the triple-gene deletion plus gC virus group than in the triple-gene deletion virus group (*, *p* < 0.05). In conclusion, the triple-gene deletion plus gC virus can elicit immunity more effectively than the triple-gene deletion virus at a lower immunization dose.

## 4. Discussion

Since 2011, newly emerging PRV epidemic strains have resulted in huge economic losses for pig farms in China [37]. Researchers found that the PRV mutant strain JS-2012 resulted in earlier and more severe clinical symptoms, as well as higher mortality, when compared with the PRV classic strain SC [38]. Based on protection tests, the Bartha-K61 vaccine provided 100% protection against the classic strain but only partially against JS-2012 and HeN1 strains [38,39,40]. However, in other studies, it was confirmed that the Bartha-K61 vaccine can protect nursery pigs and growing pigs against emerging variants of PRV, such as the HeN1 and XJ5 strains [24,41,42,43]. These conclusions may be due to differences in the age of experimental animals, immune doses, challenge doses, and challenge strains. In this study, we evaluated the efficacy of triple-gene deletion plus gC virus and triple-gene deletion virus in mice. But of all PRV-susceptible animals, the pig is more resistant than others, including the mouse. Therefore, the results in mice are not necessarily applicable to pigs. Further studies will be conducted to evaluate the efficacy of these strains in pigs.

The envelope of PRV encloses the outermost layer of the virion and has 8~10 nm spikes on its surface, which are composed of glycoproteins encoded by the virus, and are associated with the infective ability of the virus. As for the glycoproteins, gE and gI are important for the virulence and transmissibility of PRV, despite being dispensable for viral replication [44,45]. Therefore, the gE and gI genes were usually deleted to develop vaccines. The ability of viruses with defective gC genes to adsorb to cells was weakened, resulting in a low viral titer, but the virus was still infectious [46]. Studies have shown that there is no significant difference in virus release between PRV gC^−^ and PRV gC^+^ after infection, but PRV gC^−^/gE^−^ exhibits a significant release barrier in comparison to PRV gE^−^. The deletion of the gE and gI genes, as well as the mutation of the gC signal peptide, had a significant impact on the release of Bartha virus. These results indicate that the release of PRV depends on the cooperative action of gC protein and other membrane proteins. In summary, PRV gC plays a pivotal role in both the adsorption and release of viruses. Furthermore, PRV gC plays a significant role in eliciting immune responses. Hence, in order to improve immune effectiveness, the additional gC was considered to be inserted in the gE/gI deletion region. In our laboratory, an inactivated vaccine featuring the additional gC-inserted mutant (rPRV-AH-gI^−^/gE^−^/gC^+^) showed better NA titers and protection efficacy [34]. A live-attenuated vaccine is generally more efficacious than an inactivated vaccine due to the induction of T-cell-mediated immunity [27]. The TK gene is closely related to the virulence but not essential for the replication of PRV, so it can be deleted to develop a live-attenuated vaccine. On account of these features, a recombinant virus was constructed with TK/gI/gE absent and additional gC added.

In this study, the triple-gene deletion virus and the triple-gene deletion plus gC virus were constructed by homologous recombination based on the parental strain PRV-AH. The viral titers of the triple-gene deletion virus and the triple-gene deletion plus gC virus in cells were comparable to those of the parental strain, indicating that gene modification has no effect on the growth characteristics of these two recombinant viruses. However, the plaque sizes of recombinant viruses were much smaller than those of parental PRV. The heterodimer of gE/gI can facilitate the fusion of infected and non-infected cells to promote the spread of PRV, which is one of the possible reasons for the plaque reduction of gE/gI deleted viruses. The gC gene was fused with His-tag in order to confirm its insertion in the recombinant virus. The expression of the inserted gC was detected by Western blot using monoclonal antibodies against His-tag. Three forms existed in infected cells, owing to the varying degrees of glycosylation, which is consistent with the present study. In order to assess the total gC expression level, we analyzed the level of gC mRNA expression by RT-qPCR. The results showed that the level of gC expression in the triple-gene deletion plus gC strain was significantly higher compared to that in the triple-gene deletion strain or in the parental strain.

In animal experiments, PRV-specific neutralizing antibodies were induced in immunized groups, demonstrating a significant increase in titers following booster immunization. The antibody titers and survival rates of mice in the triple-gene deletion plus gC virus groups were higher than those in the triple-gene deletion virus groups with the same dosage, indicating that the insertion of additional gC could enhance the immune response of the recombinant viruses by enhancing the expression of the gC protein. However, there was no significant difference in the total IgG antibody levels among the sera of the immunized groups, likely due to the stimulation of multiple PRV proteins. In subsequent work, anti-gC-specific IgG antibodies may be detected to validate the effect of additional gC insertion. Moreover, further pig experiments will include measures of the daily weight gain, clinical score, tissue viral load, and viral shedding after challenge to evaluate the efficacy of the triple-gene deletion plus gC virus and the triple-gene deletion virus.

An immune response will be triggered when the viruses invade the host. Undifferentiated CD4^+^ T cells are stimulated to produce different T cell subsets activated by antigen-presenting cells, including Th1, Th2, Th17, and Treg cells. These immune cells play a key role in antiviral immunity by producing different cytokines. In this study, IFN-γ and IL-4 were measured as two representative cytokines. Compared with the DMEM control group, all groups immunized with vaccines induced higher levels of IFN-γ and IL-4. The highest levels of IFN-γ and IL-4 were in the triple-gene deletion plus gC virus group with 10^6.0^ TCID_50_, which indicates that enhancing gC expression facilitated the production of IFN-γ and IL-4. IFN-γ can promote the differentiation of quiescent CD4^+^ T cells into Th1 cells, increase the secretion of Th1 cytokines, and improve cellular immune function. IFN-γ plays an important role in stimulating a PRV-protective immune response [47,48]. As a Th2 cytokine, IL-4 can activate B cells and facilitate the proliferation of T cells [49]. Therefore, higher levels of IFN-γ and IL-4 induced by the triple-gene deletion plus gC virus are beneficial to specific humoral immunity and cell-mediated immunity, which is consistent with the results of neutralizing antibodies and survival rate.

## 5. Conclusions

In summary, the triple-gene deletion plus gC virus and the triple-gene deletion virus were constructed successfully in this study. The immune efficacy of the live-attenuated vaccine was significantly increased by inserting additional gC into the gE/gI deletion region, indicating that the triple-gene deletion plus gC virus is a promising candidate for attenuated vaccines to control the new-emerging PRV variants.

## Figures and Tables

**Table 1:** Primers used in this study.

Name of Primer	Sequence of Primer
TK-UA-Forward	CCGGAATTCACGTCGTTCTTGGCGATCTG
TK-UA-Reverse	TGTCCGTGTCGAACAGAGTGC
TK-DA-Forward	AAACTGCAGGATATCGCCTTCACGTCGGAGATGG
TK-DA-Reverse	CCCAAGCTTCTCGGCGGAGATGATGACC
gE-UA-Forward	CCGGAATTCACCAGCACCGCACGTACAAGTT
gE-UA-Reverse	CAGCAGCGTCCCGTCTATCGT
gE-DA-Forward	AAACTGCAGGATATCCGGAAGTGACGAATGG
gE-DA-Reverse	CTCGGTGGTGATGTAGAAAAGCTTGGG
EGFP-Forward	AACGATATCGTTTAAACGTTCTTTCCTGCGTTATCC
EGFP-Reverse	AACGATATCAACCCTATCTCGGTCTATTCT
gC-Forward	CCAAGCTTTTAAATCCGTTTCCTG
gC-Reverse	CGGGATCCCCCGACGACCAATA
TKdet-Forward	CAGGCGTTCGTAGAAG
TKdet-Reverse	GGGATGACATACACATGGC
gEdet-Forward	CGTGAACATCCTCACCGACTTC
gEdet-Reverse	GGTCAAACGTGTCCATGTCG

## Data Availability

The data presented in this study are available on request from the corresponding author. Data is contained within the article.
